# What triggers inflammation in COVID-19?

**DOI:** 10.7554/eLife.76231

**Published:** 2022-01-20

**Authors:** Tomohiro Sawa, Takaaki Akaike

**Affiliations:** 1 Department of Microbiology, Graduate School of Medical Sciences, Kumamoto University Kumamoto Japan; 2 Department of Environmental Medicine and Molecular Toxicology, Tohoku University Graduate School of Medicine Sendai Japan

**Keywords:** SARS-CoV-2, COVID-19, spike protein, cytokine storm, TLR2, inflammation, Viruses

## Abstract

The spike protein of SARS-CoV-2 triggers macrophages and epithelial cells to produce excess levels of pro-inflammatory molecules, which can do more harm than good.

**Related research article** Khan S, Shafiei MS, Longoria C, Schoggins JW, Savani RC, Zaki H. 2021. SARS-CoV-2 spike protein induces inflammation via TLR2-dependent activation of the NF-κB pathway. *eLife* **10**:e68563. doi: 10.7554/eLife.68563

It is almost two years since the start of the COVID-19 pandemic, caused by the severe acute respiratory syndrome coronavirus 2 (SARS-CoV-2). As of today, there have been more than 290 million confirmed cases and 5 million related deaths worldwide (Johns Hopkins Coronavirus Resource Center, 2022). Although SARS-CoV-2 vaccines, including those that use mRNA, have been successfully rolled out in many countries, effective treatments for severe COVID-19 are still urgently needed.

Each SARS-CoV-2 viral particle consists of a protein envelope that contains its single stranded RNA genome, which codes for four structural proteins: the spike, the membrane, the envelope, and the nucleocapsid (Yang and Rao, 2021). The spike protein binds to a receptor called angiotensin-converting enzyme 2 (ACE2) on the surface of the human epithelial cells that line the lungs. This allows the virus to enter the cell and use the cell’s RNA and protein synthesis machinery to replicate itself (Figure 1A).

**Figure 1.**
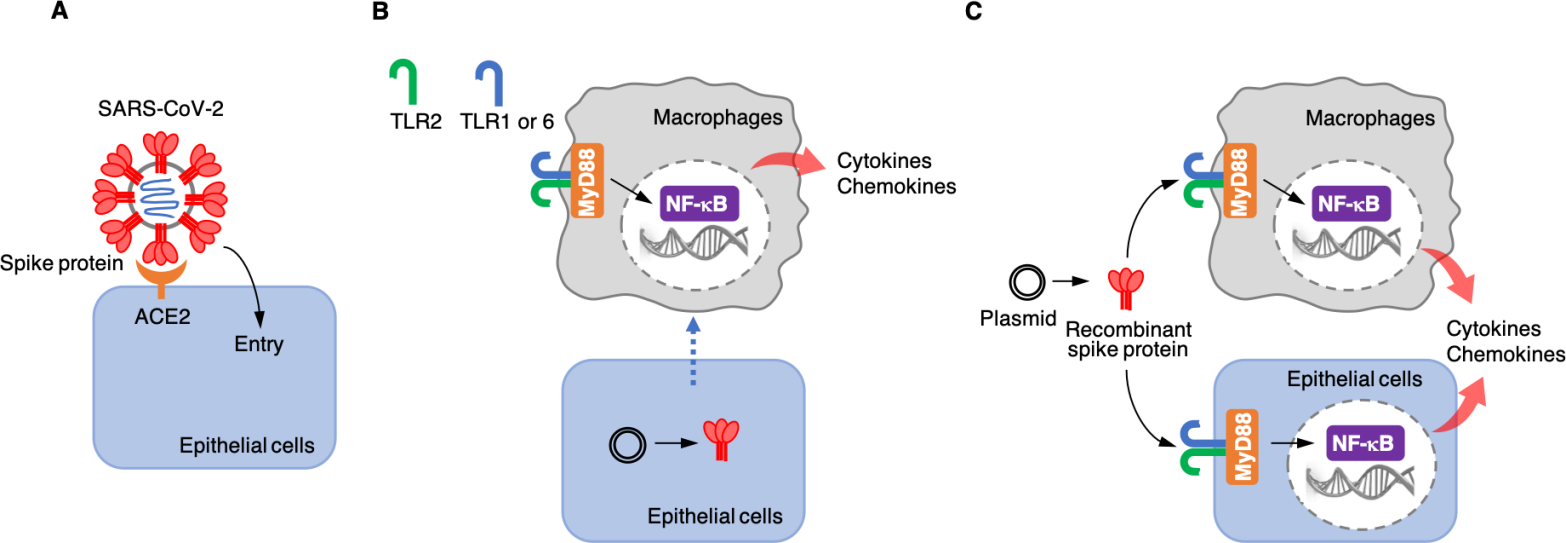
Roles of the SARS-CoV-2 spike protein during infection and inflammation. (**A**) The spike protein (red) binds to the angiotensin-converting enzyme 2 (ACE2, orange) on the surface of epithelial cells, leading to the virus entering the cells. (**B**) Khan et al. artificially introduced a plasmid containing the DNA sequence for the spike protein to epithelial cells (bottom) which were cultured together with macrophages (top) in the laboratory. This caused the epithelial cells to make the spike protein, which triggered the macrophages to produce pro-inflammatory cytokines and chemokines. However, under these conditions, the spike protein was not detected in the culture medium, suggesting that the macrophages are somehow able to sense the protein either inside or on the surface of epithelial cells. This activation requires the spike protein to bind to Toll-like receptors (TLRs) that have formed dimers – either TLR2 with TLR1, or TLR2 with TLR6. Adaptor protein MyD88 then activates a transcription factor, nuclear factor-κB (NF-κB), which induces the transcription of pro-inflammatory molecules. (**C**) Khan et al. also used a plasmid to produce recombinant spike protein in the laboratory, and then applied these proteins to the medium in which macrophages and epithelial cells were growing. This showed that the spike protein can trigger both types of cells to produce pro-inflammatory cytokines and chemokines. This activation also required the TLR dimers and MyD88.

Once the body recognizes that these viral proteins are pathogenic, it activates various immune cells, including macrophages (Amarante-Mendes et al., 2018). These immune cells produce pro-inflammatory molecules called cytokines and chemokines, which usually help the body clear the viral infection. However, if their release is poorly regulated, this can lead to a cytokine storm which can severely damage the body’s tissues and organs (Fajgenbaum and June, 2020; Blanco-Melo et al., 2020). Understanding how SARS-CoV-2 proteins activate intense inflammatory responses at the molecular level is necessary to develop treatment strategies for severe COVID-19. Now, in eLife, Hasan Zaki and colleagues from the University of Texas Southwestern Medical Center – including Shahanshah Khan as first author – report that the spike protein of SARS-CoV-2 causes potent inflammatory responses in macrophages and epithelial cells (Khan et al., 2021).

Khan et al. first studied whether any of the four structural proteins of SARS-CoV-2 could activate inflammatory responses in human macrophages. To do this, they first produced recombinant versions of the proteins in the laboratory, and then co-cultured each of the proteins with macrophages. Of the four proteins, only the spike protein triggered the production of pro-inflammatory cytokines and chemokines in a way that depended on dose and time.

Next, Khan et al. wanted to know whether epithelial cells, such as the ones that line the lung, can activate macrophages when they are infected with SARS-CoV-2. This is possible because the virus – when it infects epithelial cells – releases its RNA genome inside these cells, including the part that encodes for the spike protein. The cells may then start producing this protein and releasing it into the extracellular space or presenting it on their surface. To test whether the spike protein could activate inflammatory responses when it was expressed by epithelial cells, Khan et al. grew epithelial cells in the lab, introduced DNA coding for the spike protein into them, and co-cultured them with macrophages. The experiment showed that the macrophages produced pro-inflammatory cytokines (Figure 1B), but the spike protein was not found in the medium used to grow the cells. These results suggest that infected epithelial cells do not release the spike protein into the extracellular environment, and that macrophages instead somehow sense the spike proteins produced in epithelial cells through direct or physiochemical interactions.

Khan et al. then wanted to find out which part of the spike protein was responsible for the inflammatory response. The spike protein is divided into two functionally different domains: the S1 subunit, which binds to ACE2; and the S2 subunit, which helps the virus to enter epithelial cells. Khan et al. produced each of these subunits separately in the laboratory and co-cultured them with macrophages to determine which of the domains of the protein triggered the inflammatory response. They found that both subunits were able to activate macrophages to produce pro-inflammatory cytokines and chemokines in vitro. Khan et al. also co-cultured the two spike protein subunits with epithelial cells similar to those that line the lung, which resulted in the epithelial cells also producing pro-inflammatory molecules (Figure 1C).

Interestingly, both spike protein subunits failed to induce the expression of anti-viral proteins called type I interferons (interferon-1α and interferon-β), which are part of the body’s natural defense system against disease-causing agents. This is similar to what is seen in patients with severe COVID-19, who have high levels of pro-inflammatory cytokines, but low levels of type I interferons. To demonstrate that the inflammatory responses induced by the spike protein do not depend on ACE2 binding, Khan et al. used an ACE2 inhibitor. When the inhibitor was applied to cells that had been co-cultured with the spike protein, the cells still produced cytokines and chemokines.

Finally, Khan et al. tried to determine which receptors and associated signaling pathways were required for the inflammatory responses induced by the spike protein. Human cells have various receptor proteins on their surface that can recognize viral proteins and trigger downstream signaling pathways (Amarante-Mendes et al., 2018; Zhou et al., 2021). A group of these receptors are called Toll-like receptors (TLRs). Khan et al. showed that, in order for macrophages and epithelial cells to recognize the spike protein, one of these receptors, called TLR2, needs to dimerize with TLR1 or TLR6. When the spike protein binds to one of these dimers, it activates the adaptor protein MyD88, which in turn activates nuclear factor κB, a transcription factor that regulates the expression of pro-inflammatory cytokines and chemokines (Figure 1C).

A recent study reported that expression of TLR2 and MyD88 was associated with COVID-19 disease severity (Zheng et al., 2021). This, combined with the results of Khan et al., suggests that TLR2 and its downstream signaling pathways may be good therapeutic targets for attenuating the cytokine storm and improving the survival of patients with COVID-19.
